# Investigating the Relationship Between Hoof, Liver, and Cardiac Abnormalities in *Bos taurus* Grain-Fed Beef Cattle < 30 Months in a Natural Program with No Antibiotics or Growth Promotants

**DOI:** 10.3390/ani16203208

**Published:** 2026-10-12

**Authors:** Emma M. Hamilton, Karly N. Anderson, Kurt D. Vogel, Temple Grandin

**Affiliations:** 1 Department of Animal Sciences, Colorado State University, Fort Collins, CO 80523-1171, USA; templegrandinassistant@gmail.com; 2 Department of Animal and Food Science, University of Wisconsin–River Falls, River Falls, WI 54022, USA; karly.anderson@uwrf.edu (K.N.A.); kurt.vogel@uwrf.edu (K.D.V.); 3 Humane Handling Institute, University of Wisconsin–River Falls, River Falls, WI 54022, USA

**Keywords:** corkscrew hoof, cattle, cardiac remodeling, natural, liver abscess, welfare

## Abstract

Our primary objective was to determine the relationship between hoof, liver, and cardiac abnormalities in a group of natural-fed cattle at slaughter. *Bos taurus* beef cattle (N = 1312), without dairy phenotype, were evaluated for abnormalities at a slaughter establishment in the Great Plains region of the United States (elevation: 563 m). Cattle were enrolled in a vegetarian-fed program that prohibited implants, diet-included hormones, and beta-adrenergic agonists. Generalized linear mixed models were used to determine the prevalence of each abnormality within other abnormalities. There was no detectable difference (*p* ≥ 0.1630) to support an association between the proportion of cattle with each hoof score within each heart or liver score. Cattle with no liver abscesses were more prevalent (*p* = 0.0355) among cattle with normal cardiac morphology (Heart Score (HS)1) (94.37 ± 1.19% (mean ± SE)) than cattle with HS2 (very mild remodeling) (88.6 ± 1.42%). Further research is needed to understand this observed difference between two similar heart scores and to further explore contributing factors to these production diseases.

## Simple Summary

Production diseases include those whose etiologies relate, in part or completely, to pushing the physiology of animals to be more efficient via diet, breeding, and management. Hoof abnormalities, liver abscesses, and cardiac remodeling are three production diseases that are present in fed cattle today. The aim of this study was to determine if relationships exist between these production diseases and if relationships exist between each disease and selected carcass characteristics in a group of cattle in one natural feeding program. The results of this study yielded no evidence of an association between hoof abnormalities and liver abscesses or hoof abnormalities and cardiac remodeling. There was evidence to support a relationship where more cattle with no liver abscesses had a greater prevalence of normal cardiac morphology than very mild cardiac remodeling. More cattle with severe liver abscesses were graded Select than Prime or Choice. Cattle with shovel hooves had a lighter hot carcass weight than cattle with normal hooves. Further relationships existed between hoof abnormalities, liver abscesses, cardiac remodeling, and select carcass characteristics. These findings warrant further evaluation of genetic variables influencing phenotypic expression of these production diseases.

## 1. Introduction

There are 25 million fed steers and heifers slaughtered each year in the United States [1]. Thirty years ago, there were 27 million cattle on feed slaughtered each year [2]. Despite the decrease in the total number of cattle slaughtered, total pounds of production increased from 11 billion kg in 1996 to 12 billion kg in 2024 [2]. During this time, cattle size, growth, and efficiency have increased [3,4,5,6]. This has been accomplished primarily in two ways: (1) genetic selection for growth [6], muscling, and efficiency, and (2) development in feeding and production programs to increase growth of the carcass [7]. Growth promotants such as fed ionophores, growth hormone implants, and fed beta-adrenergic agonists are three tools that can increase cattle growth on the feedyard [8]. The addition of these factors has yielded larger and faster-growing cattle on feedyards today.

The fed cattle industry can be separated into three main feeding types: conventional, natural, and organic. Conventional cattle have no restrictions on diet, administration of vaccines or antibiotics, or other supplemental growth promotants [9]. All of these technologies that are used, however, must be used by label directions and follow their respective withdrawal times. Natural cattle consist of animals enrolled in a variety of programs [10]. The blanket term “natural” does not indicate one set of characteristics for an animal but typically indicates some limitation of antibiotics and hormone administration [10]. The USDA Process Verified Program lists several programs that fall under the “natural” category, with most program requirements being that the animals are fed a vegetarian diet, raised without the use of growth-promoting hormones, and with responsible use of antibiotics [11]. The USDA lists a standard for organic program cattle which must follow several requirements including, but not limited to, being produced without genetic engineering, allowed year-round access to outdoors, raised on organic land, fed 100% organic feed, and managed without antibiotics, added growth hormones, or animal by-products [12].

In 2005, Rollin (2005) introduced the concept of production diseases, which arose from pushing the physiology of animals to grow faster, larger, and more muscular beyond their biological limits [13]. Three production diseases that are present in fed cattle today include hoof abnormalities, liver abscesses, and cardiac remodeling [14].

Hoof abnormalities are any change in hoof conformation that deviates from normal, such as a hoof with a short, wide toe, high heels, and both claws the same length and even in size [15]. Hoof abnormalities result from genetics, consequences of high-concentrate diets such as laminitis, and damage caused by environmental factors [16,17]. Hoof abnormalities are a concern for the industry. In dairy cattle, hoof abnormalities have the capacity to contribute to 90% of incidences of lameness [18]. In beef cattle, hoof abnormalities have been reported to be low to moderately heritable in certain breeds [19]. Lameness is a painful disorder impacting locomotion and mobility in cattle [20,21]. In general, hoof abnormalities can impact animal behavior, cause pain, and contribute to the incidence of nonambulatory cattle at slaughter [22].

Liver abscesses result from subacute ruminal acidosis (SARA), a condition brought on by high-concentrate diets with low levels of forage [23]. These diets are prevalent in the fed-cattle industry because feedstuffs such as steam-flaked corn cause cattle to have a higher average daily gain and add more fat during the feeding phase, both of which are rewarding to the producer and slaughter establishment [6,24]. A liver abscess is characterized as a bacterial infection, and thus, all livers with abscesses must be condemned [25,26]. In 2023, this resulted in a $61.2 million loss to the industry [27]. In the past 30 years, the prevalence of liver abscesses has gone from 17.8% to 17.5% [3,5]. Despite several research efforts to reduce the prevalence of liver abscesses, the issue still has not decreased.

Congestive heart failure (CHF) is the result of right-sided cardiac remodeling due to vasoconstriction of pulmonary blood vessels to account for hypoxic conditions in the lungs [6,28]. These hypoxic conditions historically caused CHF at high altitudes, but in the past 10 years, several studies have reported right-sided cardiac remodeling in cattle at slaughter and death of cattle on feedyards resulting from CHF [14,29,30]. Kukor et al. (2021) [30] reported that sire can have a significant effect on heart structure, indicating a genetic role in cardiac remodeling. Vogel et al. (2020) [31] measured pulmonary arterial pressure (PAP) in fed steers that were either administered a growth implant (Revalor) or no implant and reported that there was no difference in PAP score between the cattle that were administered an implant and those that were not. It is not known if further dietary components or feed additives have an impact on an animal’s potential to develop CHF at the time of this study.

Although hoof abnormalities, liver abscesses, and cardiac remodeling are all production diseases which are present in the fed-cattle industry today, it was not known if there would be existing relationships between the three diseases. Some hoof abnormalities, such as shovel (long) hooves and corkscrew claws, can result from laminitis, a consequence of SARA with a shared etiology to liver abscesses [32,33,34]. Cardiac remodeling may be a result of increased cattle size [6,35], which is also associated with specific hoof abnormalities [16]. In a study evaluating all three of these production diseases in conventional cattle, there was no evidence to support a relationship between either disease [14]. There is a lack of research evaluating the impact of growth-promoting technologies on production diseases. It is possible that relationships between production diseases could exist in natural-fed cattle whose physiological systems are not impacted by growth-promoting technologies. The objective of this study was to determine whether relationships exist between hoof abnormalities, liver abscesses, and right-sided cardiac remodeling in a group of natural-program-fed cattle. A secondary objective was to determine whether relationships exist between those three production diseases and relevant carcass characteristics.

## 2. Materials and Methods

The cattle observed in this study were sourced from five commercial feedyards and were not manipulated by the data collection team in any way. Cattle were slaughtered in a federally inspected slaughter establishment under the Humane Methods of Slaughter Act (7 USC 1901) (United States Electronic Code of Federal Regulations, 2026) [36] and the regulations that enforce it (9 CFR 313) (United States House of Representatives Office of the Law Revision Counsel, 2026) [37] under inspection by the USDA Food Safety and Inspection Service (FSIS). All cattle were rendered insensible and exsanguinated before data collection procedures occurred. Therefore, an IACUC exemption protocol was submitted and approved by Colorado State University (7464) on 10 November 2025.

The study conducted was an observational study where data was collected at a high-speed slaughter establishment in January of 2026. All animals were observed at a single slaughter establishment in the Great Plains region of the United States (elevation: 563 m (FAA, 2023)). Cattle were sourced from five producers (Producer A: n = 244, Producer B: n = 5, Producer C: n = 31, Producer D: n = 957, Producer E: n = 75) and enrolled in the same natural program, which prohibits the use of antibiotics, added hormones, beta-adrenergic agonists, or ionophores. Cattle were of Angus heritage and non-dairy influence. Inclusion requirements at data collection required that cattle be of *Bos taurus* beef-type and <30 mos of age. A total of N = 1312 steers were enrolled in this study (HCW = 464.85 ± 53.44 kg (mean ± SD)). The specific breed, origin of each animal, backgrounding program, and specific feed and vaccination protocol were not available to the data collection team, as all cattle were observed after transfer of custody to the collaborating slaughter establishment.

R Studio Version 4.4.1 was used to conduct a power calculation using power.prop.test with a conjectured sample size of 1000, an expected proportion of 14% of animals without cardiac remodeling to have liver abscesses, and an expected proportion of 21% of animals with cardiac remodeling to have liver abscesses, based on anecdotal observations from previous work. This yielded a power of 0.83 (83%). Therefore, a target of 1000 animals was set for data collection in order to conduct a robust study to determine the relationship between these production diseases. The data collection team observed all of the animals that were processed during the first half of A shift at the collaborating slaughter establishment, which was estimated to meet the total number of animals needed for the power calculation. Due to the rapid line speed and proximity of data collection team members to USDA inspectors, some liver and heart scores were missed. Following data collection, all cattle without all three scores were excluded from the study, yielding a final sample of N = 1312 animals.

A hoof scorecard developed by Hamilton et al. (2026) was used to evaluate hoof conformation [14]. The front two hooves of each animal were scored following exsanguination while the animals were shackled, and before hide removal. All hoof scores that were present on either of the front two hooves were recorded. For some animals, one hoof had two conformation types. Therefore, the final hoof score for each animal was a combination of all of the scores that were present. Each animal earned one score, which could be one conformation type or a combination of conformation types. These combinations, when observed, counted as their own separate hoof scores (i.e., hoof WI). The same observer that recorded hoof score recorded hide color for each animal for descriptive statistics. Heart and liver scorers were blinded to the hoof and hide score for each animal.

Liver abscesses and hearts were scored after evisceration on the organ table. One observer recorded liver score and carcass ID, and another observer recorded heart score and carcass ID. A liver abscess scorecard developed by Hamilton et al. (2026) [14] based on the Elanco liver scoring system [38] was used to score each liver for each animal. The heart scoring system was developed by Holt (2020) [39] and previously used by Heffernan et al. (2020) [40]. Hoof, liver, and heart scorers were blinded to other scores. The hoof and liver scoring personnel were trained using approximately 40 unique pictures of each respective condition. After two weeks of training, there was a randomized test to confirm that each observer could identify the condition and its severity with a minimum agreement of 80% between the observer and the group of coauthors (EMH, KNA, KDV) who developed the randomized test; 80% agreement was determined by dividing the number of scores the observer correctly identified by the total number of images included on the randomized test. The personnel scoring hearts was a trained observer on the Holt (2020) [39] scorecard who scored for multiple previous unrelated studies.

**Figure 1:**
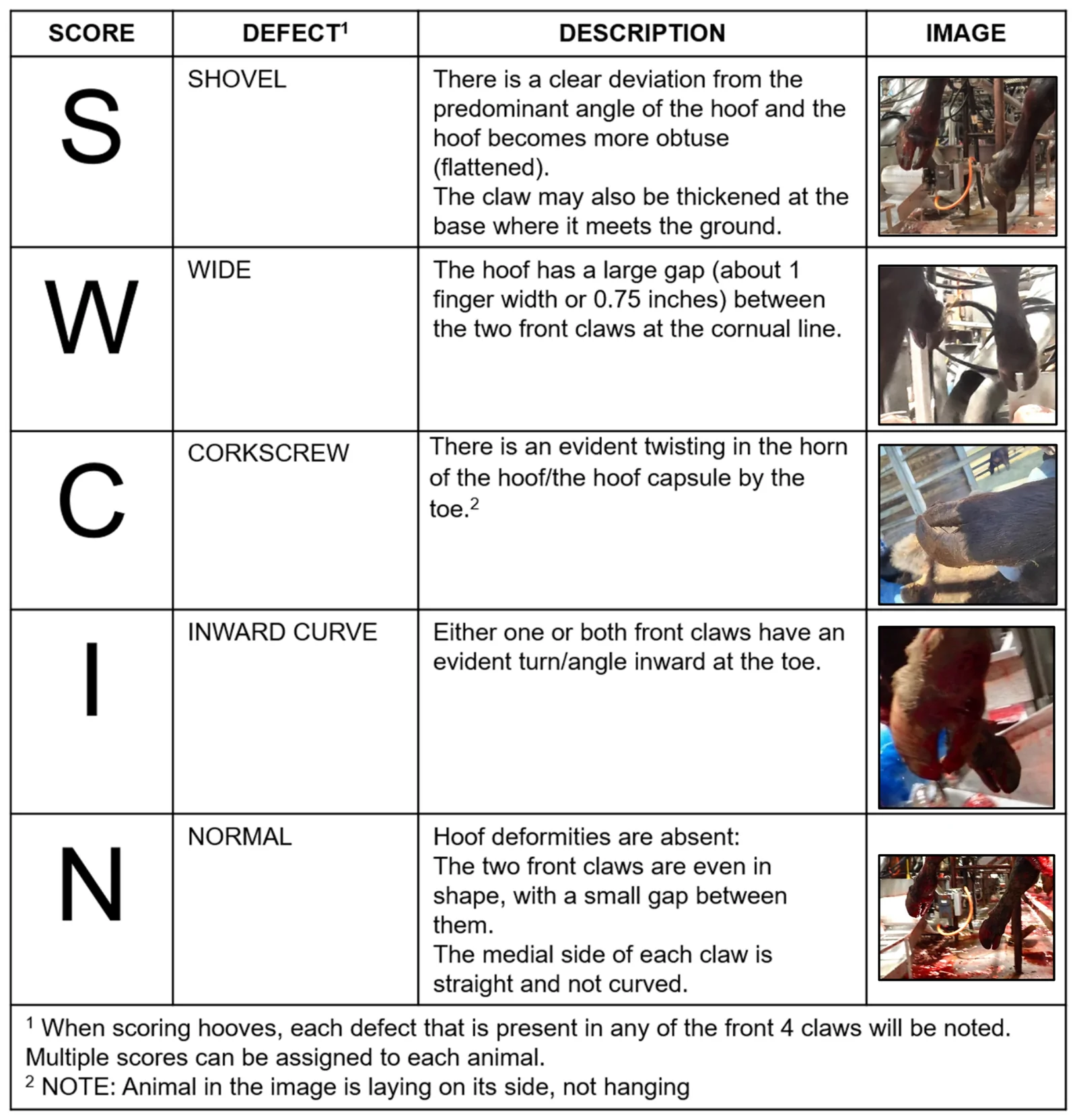
Bovine Hoof Conformation Scorecard courtesy of Hamilton et al. (2026) [14].

The hoof scorecard depicted in Figure 1 consisted of a 5-point categorical scale with four scores for abnormal hooves and one score for normal [14]. The abnormal scores consisted of HOOF S (shovel hoof), which was a hoof with an elongated toe, often flattened at the base; HOOF I (inward curve), which was a hoof where one or both toes pointed inward; HOOF C (corkscrew), which was the severe form of HOOF I where the toe horn was curling inward or crossed over the other toe; and HOOF W (wide toe), where there was a large gap between the interdigital cleft. A HOOF N (normal hoof) was defined as a hoof where both front toes were evenly shaped with a small space between the interdigital cleft. To earn a score of HOOF N (normal), both front hooves had to be free of abnormalities.

**Figure 2:**
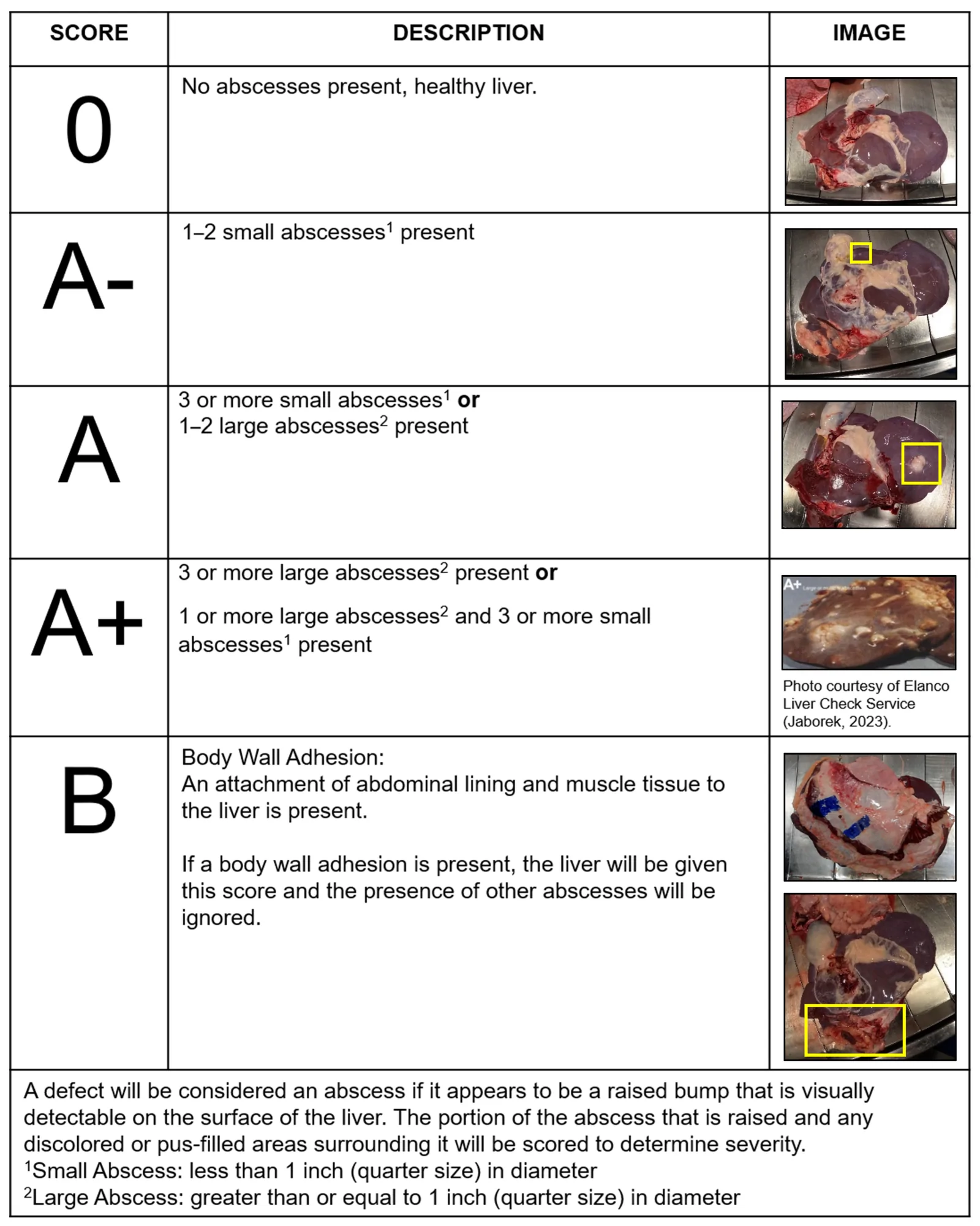
Bovine Liver Abscess Scorecard courtesy of Hamilton et al. (2026) [14,27].

The liver abscess scorecard depicted in Figure 2 consisted of five different liver scores [14]. A LIVER 0 (no abscess) indicated that there were no abscesses present on the liver. LIVER A- indicated that 1–2 small (less than 6.35 mm in diameter) abscesses were present. LIVER A indicated that 1–2 large (greater than 6.35 mm in diameter) abscesses were present or 2–3 small abscesses. LIVER A+ indicated that 2 or more large abscesses were present with 2 or more small abscesses [27]. LIVER B indicated that the liver was adhered to the body wall, with evidence of that adhesion present on the liver.

**Figure 3:**
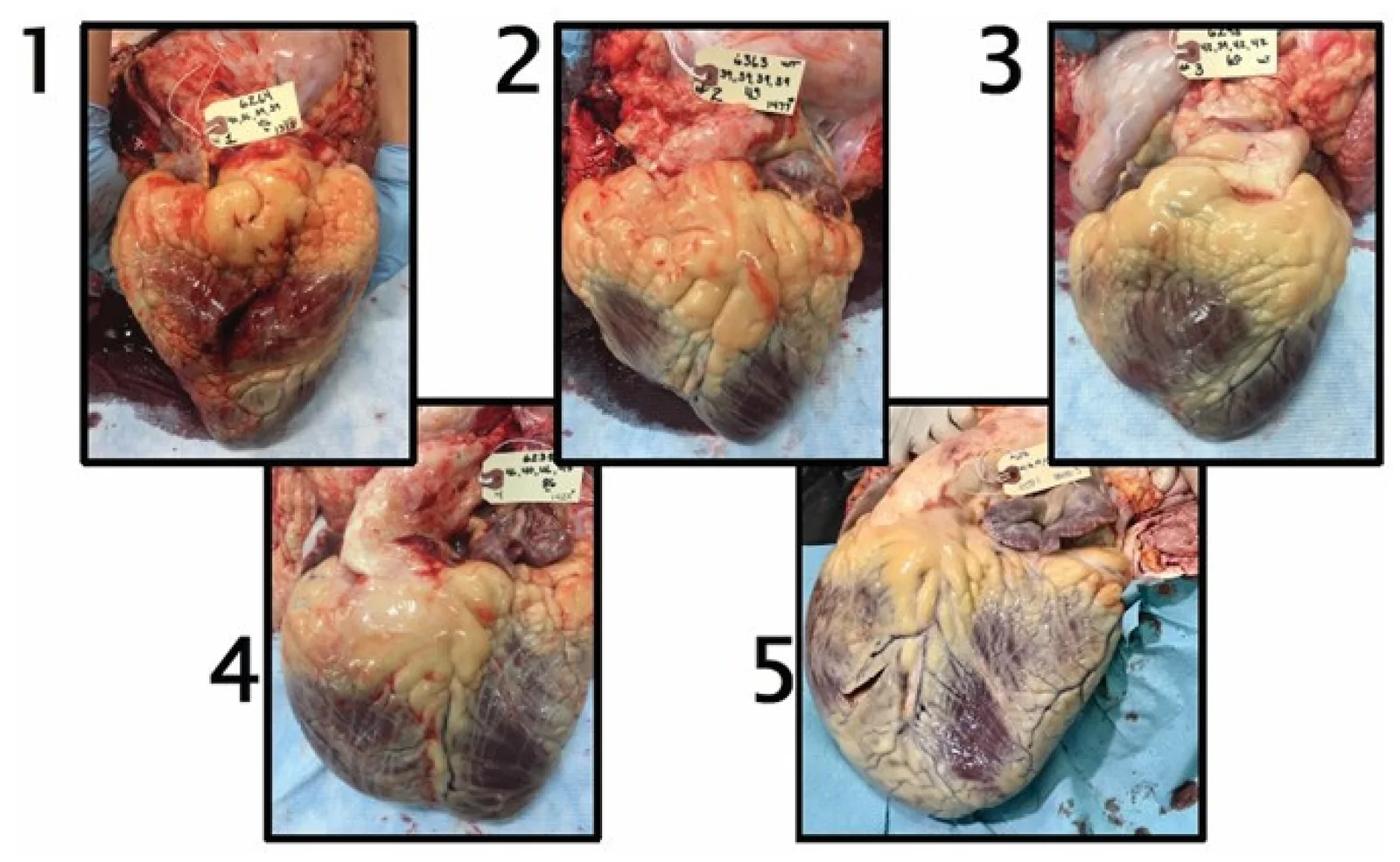
Bovine Heart Scorecard courtesy of Heffernan et al. (2020) [40].

The heart scorecard depicted in Figure 3 consisted of five different heart scores, ranging from 1–5. A score of HS1 indicated a normal heart with a normal conical shape, normal atrial anatomy, and no clinical evidence of cardiac remodeling [39]. Heart Score 2 (HS2) indicated mild change, where the right atrium and ventricle are beginning to become larger than the left, and the pulmonary artery is beginning to show mild enlargement [39]. Heart Score 3 (HS3) indicated moderate changes, where the heart is beginning to lose its conical shape, the right ventricle is larger than the left and becoming more pronounced, and there is evident thinning of the vessel wall [39]. Heart Score 4 (HS4) indicated severe changes with loss of cardiac conical shape, the left ventricle apex is lost due to ventricular rounding, and the right atrium is enlarged and congested [39]. Heart Score 5 (HS5) indicated severe changes and a flaccid heart, where the left ventricle apex is lost, the right ventricle and atrium are large and round, and the cardiac muscle is soft and without shape [39].

Hoof, liver, and heart scores were recorded at slaughter. After data collection, all carcasses continued through normal slaughter establishment procedures where carcass data was recorded. Hot carcass weight was recorded from a digital scale. Quality Grade (QG), Yield Grade (YG), Ribeye Area (REA), and Fat Thickness (FT) were recorded from a standard imaging device used in normal operating procedures (e + v Standard Grading Camera, e + v Technology, GmbY & Co. KG, Brandenburg, Germany). Final USDA QG and YG were confirmed by in-person USDA inspectors for grading, and these grades were used for final data analysis.

A convenience sample of cattle, informed by a statistical power calculation, from one natural-fed program was evaluated for this cross-sectional observational study. Hoof score, hide color, liver score, and heart score were all matched to individual carcass ID, and post-mortem carcass characteristics provided by the slaughter establishment were matched to each respective carcass ID for analysis. During data collection, there was a malfunction with the grading camera for some carcasses for REA (n = 24) and FT (n = 366), so those carcasses were not included in the analysis for those respective characteristics. Three animals displayed *Bos indicus* characteristics and were removed from the study. This yielded a final total of N = 1312 *Bos taurus* beef-type steers.

Prior to statistical analysis, all measurements were converted to metric units. Pivot tables within Microsoft Excel were used to determine the proportion of animals within each hoof, liver, and heart score. The *binom* package within R version 4.4.1 and R Studio version 2024.04.2+764 [41] was used to report proportion, percent, and 95% confidence intervals (CI) for each hoof, liver, and heart score.

To assess the primary objectives of this study, the GLIMMIX procedure within SAS Enterprise Guide 7.1 (Statistical Analysis Systems Institute, Inc., Cary, NC, USA) was used to perform comparisons between the proportion of cattle with each production disease score present within other production disease scores and with each carcass characteristic (USDA QG and USDA YG) present within each production disease score. For all comparisons between groups, the hoof scores that were observed in all of the cattle were combined into the following base categories from the scorecard: HOOF C (C, CW), HOOF I, HOOF N, HOOF S (S, SC, SI, SWI), HOOF W, and HOOF WI. HOOF W and HOOF WI were kept in separate categories because Hamilton et al. (2026) [14] observed differences between each of those scores in their own category compared to other scores and sample size for each score was sufficient (n > 5). Liver scores consisted of the following categories: LIVER 0, LIVER A-, LIVER A, and LIVER A+/B. Liver scores A+ and B were combined due to underrepresentation of cattle with LIVER B and their shared characteristics of having severe liver abscesses. Models were developed within the GLIMMIX procedure that included the link = logit function and Satterthwaite adjustment for binomially distributed data to produce proportions for each production disease score. The animal was considered the experimental unit. Denominator degrees of freedom were calculated using the Satterthwaite method. Dependent variables included heart score, hoof score, or liver score, and only one dependent variable was considered for each model. Producer was included as a categorical covariate to account for potential cattle source effects, and means were reported from each model when the effect was significant (*p* < 0.05) [42,43]. To control for multiplicity, a Tukey–Kramer post hoc adjustment was applied within all models for binomially distributed data.

SAS Enterprise Guide 7.1 (Statistical Analysis Systems Institute, Inc.) was used to perform the UNIVARIATE procedure with the histogram statement for each continuous variable (HCW, REA, FT) for each individual score present (within hoof, liver, and heart scores). Two out of the three following conditions needed to be met for a variable to be considered normal: the histogram was normally distributed, the Cramer–von Mises *p*-value was >0.05, and the quantile plot appeared normal [44]. The following results were identified as not normally distributed and therefore not included in comparisons between means: REA for HOOF S and FT for HOOF S.

To assess the secondary objectives of this study, the MIXED procedure within SAS Enterprise Guide 7.1 (Statistical Analysis Systems Institute, Inc.) was used to perform Student’s *t*-tests with the Tukey–Kramer adjustment to determine differences in means for all normally distributed continuous variables (HCW, REA, FT) between each score for categorical variables (hoof, liver, and heart score). The model was run with producer as an interaction effect. If the interaction was significant, it was included in the final model to compare means. If the interaction was not significant, a model without the interaction effect was used to compare means between score categories. For all comparisons between means, categories with n < 5 were not included in comparisons due to underrepresentation in the observed categories. Those groups are reported in each respective table.

## 3. Results

### 3.1. Feedlot Cattle Sample

Descriptive statistics for the 1312 steers in this study are reported in Table 1. Average hot carcass weight (HCW) for this group of animals was 464.85 ± 53.44 kg (mean ± SD). Average ribeye area (REA) for this group (n = 1288) was 87.15 ± 9.35 cm^2^. Average FT for this group (n = 946) was 3.93 ± 0.54 cm. Sixty-one percent (807/1312) of cattle in this study were black-hided, 12% (159/1312) had black and white hides, 8% (105/1312) had gray hides, 12% (155/1312) were red-hided, 4% (50/1312) were red and white, 2% (30/1312) were tan-hided, and 0.4% (6/1312) were tan and white.

### 3.2. Hoof Abnormalities

Fifty-seven percent (751/1312) of cattle had at least one hoof abnormality present. The proportion of cattle with each hoof abnormality type is reported in Table 2 (%, proportion, 95% CI). The proportion of cattle with HOOF C was 4.04% (53/1312, 95% CI: 3.04 to 5.25%). The proportion with HOOF I was 40.02% (525/1312, 95% CI: 37.35 to 42.72%). The proportion with HOOF S was 0.53% (7/1312, 0.21 to 1.10%). The proportion with HOOF SC was 0.15% (2/1312, 95% CI: 0.02 to 0.55%). The proportion with HOOF SI was 0.76% (10/1312, 95% CI: 0.04 to 1.40%). The proportion with HOOF SWI was 0.38% (5/1312, 95% CI: 0.12 to 0.89%). The proportion with HOOF WC was 0.08% (1/1312, 95% CI: 0.00 to 0.42%). The proportion with HOOF W was 2.59% (34/1312, 95% CI: 1.80 to 3.60%). The proportion with HOOF WI was 8.69% (114/1312, 95% CI: 7.22 to 10.35%). The proportion of cattle with HOOF N was 42.76% (561/1312, 95% CI: 40.06 to 45.49%).

### 3.3. Liver Abscesses

Nine percent (114/1312) of cattle in this study had liver abscesses. The proportion of cattle with each liver abscess score is reported in Table 2 (percent, proportion, 95% CI). The proportion of cattle with LIVER A- was 5.56% (73/1312, 95% CI: 4.39 to 6.95%). The proportion of cattle with LIVER A was 1.83% (24/1312, 95% CI: 1.16 to 2.71%). The proportion of cattle with LIVER A+ was 0.76% (10/1312, 95% CI: 0.37 to 1.40%). The proportion of cattle with a body wall adhesion (LIVER B) was 0.53% (7/1312, 95% CI: 0.21 to 1.10%). Ninety-one percent (91.31%) of cattle had no liver abscesses (LIVER 0) (1198/1312, 95% CI: 89.85 to 92.78%).

### 3.4. Heart Abnormalities

Thirty-four percent of cattle in this study had signs indicating moderate to severe cardiac remodeling (HS3 and HS4). The proportion of cattle with each heart score is reported in Table 2 (percent, proportion, 95% CI). Twenty-eight percent (28.43%) of cattle had normal hearts (HS1) (373/1312, 95% CI: 26.00 to 30.96%). Cattle with HS2 (very mild remodeling) represented 37.73% (495/1312, 95% CI: 35.10 to 40.41%) of those in this study. Cattle with HS3 were 31.63% (415/1312, 95% CI: 29.12 to 34.22%) of those in this study. Cattle with HS4 were 2.21% (29/1312, 95% CI: 1.49 to 3.16%) of those in this study. No cattle in this study scored HS5 (severe changes with a flaccid heart).

### 3.5. Relationships Between Production Diseases

Twenty-two percent (294/1312) of cattle had two conditions present. Of those, 3.43% (45/1312) had hoof abnormalities and liver abscesses, 17.38% (228/1312) had hoof abnormalities and cardiac remodeling, and 1.60% (21/1312) had liver abscesses and cardiac remodeling present. Sixteen animals had all three conditions, representing 1.22% of the sample population. The proportion of cattle with multiple abnormalities present is reported in Table 3.

There was no evidence (*p* ≥ 0.1630) to support a difference in the proportion of cattle with each hoof score within each liver score. All data presented are estimated marginal means (observed proportion). Cattle with normal hooves consisted of 35.32 ± 4.78% (mean ± SE) of those with no liver abscesses, 54.45 ± 11.65% of those with LIVER A-, 39.43 ± 12.67% of those with LIVER A, and 34.68 ± 7.14% of those with liver A+/B. The proportion of cattle with each hoof abnormality within each liver abscess score is reported in Table 4.

The proportion of animals with each hoof score within each heart score is reported in Table 5. All data presented are estimated marginal means (observed proportion). There was no evidence (*p* ≥ 0.2212) to support a difference in the proportion of animals with each hoof score within each heart score. Cattle with normal hooves consisted of 35.06 ± 5.07% of those with no cardiac remodeling (HS1), 34.44 ± 5.08% of those with HS2, 36.51 ± 5.23% of those with HS3, and 44.22 ± 10.44% of those with HS4.

Cattle with normal livers (LIVER 0) comprised a significantly greater (*p* = 0.0043) proportion of animals with normal hearts (HS1) (94.37 ± 1.19%) than those with mild cardiac remodeling (HS2) (88.69 ± 1.42%). There was no evidence (*p* ≥ 0.1288) to support a difference in the proportion of cattle with normal livers comprising HS1 between those with HS3 (91.57 ± 1.36%) and HS4 (93.10 ± 4.71%). The proportion of animals with each liver abscess score comprising each heart score is reported in Table 6. All data presented are estimated marginal means (observed proportions). There was no evidence (*p* ≥ 0.1119) to support a difference in cattle with liver scores A-, A, and A+/B between heart scores.

### 3.6. Relationships Between Hoof Conformation and Carcass Characteristics

Average HCW, REA, and FT for each hoof score are reported as means ± SE in Table 7. Cattle with HOOF S (435.99 ± 10.87 kg) tended (*p* = 0.0632, 0.0588) to have lower HCW than cattle with an inward curve (HOOF I) (466.79 ± 2.33 kg) or cattle with a normal hoof (HOOF N) (467.04 ± 2.25 kg).

There was no evidence (*p* ≥ 0.4058) to support a difference in HCW between cattle with HOOF S, HOOF I, and HOOF N and those with HOOF C (456.92 ± 7.25 kg), HOOF W (452.61 ± 9.14 kg), or HOOF WI (458.66 ± 5.00 kg). The REA and FT for cattle with HOOF S did not meet normal distribution requirements for statistical analysis and were excluded from comparisons between means. There was no evidence (*p* ≥ 0.4480) to support a difference in average REA or FT for each remaining hoof score (HOOF C, HOOF I, HOOF N, HOOF W, HOOF WI).

The mean proportion of cattle with each hoof score within each USDA QG is reported in Table 8 as mean ± SE. All data presented are estimated marginal means (observed proportion). There was no evidence (*p* = 0.1464) to support a difference in the proportion of cattle with normal hooves within each USDA QG category. Cattle with corkscrew hooves comprised significantly less (*p* = 0.0061) of the Prime category (2.94 ± 0.57%) than the Choice category (6.88 ± 1.26%). No cattle with corkscrew hooves were graded select (0.00 ± 0.00%). There was no evidence (*p* ≥ 0.2670) to support a difference to support a difference in the proportion of cattle with each remaining hoof score (HOOF I, HOOF S, HOOF W, HOOF WI) between each USDA QG.

The mean proportion of cattle with each hoof score within each USDA YG is reported in Table 9 as mean ± SE. All data presented are estimated marginal means (observed proportion). There was no detectable difference (*p* = 0.6610) to support an association between the proportion of cattle with normal hooves within each USDA YG category. Cattle with HOOF I comprised a greater proportion (*p* ≤ 0.0375) of YG 2 (55.17 ± 6.54%) than YG 3 (40.72 ± 2.25%) and YG 4 (36.93 ± 2.03%). Cattle with HOOF I tended (*p* = 0.0933) to comprise a greater proportion of YG 2 than YG 5 (42.72 ± 33.92%). Comparisons for HOOF I did not include YG 1 (0.00 ± 0.31%) due to underrepresentation. For the remaining hoof scores (HOOF C, HOOF S, HOOF W, and HOOF WI), there was no detectable difference (*p* ≥ 0.7131) to support an association between the proportion of each hoof score comprising each USDA YG.

### 3.7. Relationships Between Liver Abscesses and Carcass Characteristics

Average HCW, REA, and FT for each liver score are reported as means ± SE in Table 10. There was a significant producer interaction (*p* = 0.0335) for HCW and liver score; however, due to underrepresentation, means are only reported for LIVER 0 and are not reported for liver scores A-, A, or A+/B. The average HCW for cattle with no liver abscesses was 448.24 ± 4.44 kg. There was no evidence (*p* ≥ 0.2404) to support a detectable difference in REA or FT between liver abscess scores.

The proportion of cattle with each liver score composing each USDA QG is reported in Table 8. All data presented are estimated marginal means (observed proportion). For both USDA QG and USDA YG, scores LIVER A-, LIVER A, and LIVER A+/B are excluded from analysis due to a total n < 5. There was no detectable difference (*p* ≥ 0.5548) to support an association between the proportion of cattle with LIVER 0 between USDA QG categories. The proportion of cattle with each liver score composing each USDA YG is reported in Table 9. There was no evidence (*p* ≥ 0.7737) to support a detectable difference in the proportion of cattle with LIVER 0 between each USDA YG.

### 3.8. Relationships Between Heart Score and Carcass Characteristics

Average HCW, REA, and FT for each heart score are reported in Table 11 as means ± SE. Cattle with HS3 had a greater (*p* ≤ 0.0001) HCW (476.16 ± 2.60 kg) than cattle with HS2 (460.76 ± 2.38 kg) or HS1 (457.34 ± 2.74 kg). There was no evidence (*p* ≥ 0.6291) to support a detectable difference between the average HCW for cattle with HS4 (469.54 ± 9.82 kg) and other heart scores. Cattle with HS2 had a significantly smaller (*p* ≤ 0.0430) REA (86.06 ± 0.43 cm^2^) than cattle with HS1 (87.81 ± 0.49 cm^2^) or HS3 (87.71 ± 0.46 cm^2^). There was no detectable difference (*p* ≥ 0.3966) to support an association for REA between cattle with HS2 and cattle with HS4 (88.85 ± 1.73 cm^2^), or between HS1, HS3, and HS4. Cattle with HS4 tended (*p* = 0.0883, 0.0959) to have less FT (3.69 ± 0.11 cm) than cattle with HS2 (3.96 ±0.03 cm) and HS3 (3.96 ± 0.03 cm), respectively, but there was no evidence (*p* = 0.4297) to support a detectable difference from HS1 (3.87 ± 0.03 cm). There was no detectable difference (*p* ≥ 0.1712) to support an association for FT between cattle with HS2, HS3, or HS1.

The proportion of cattle with each heart score within USDA QG is reported in Table 8. All data presented are estimated marginal means (observed proportion). There was no detectable difference (*p* ≥ 0.3453) to support an association between the proportion of cattle with each heart score within each USDA QG.

The proportion of cattle with each heart score within each USDA YG is reported in Table 9. All data presented are estimated marginal means (observed proportions). Cattle with HS1 comprised 57.08 ± 8.17% of the animals within YG2, which is not different (*p* = 1.000) from those within YG 4. There was evidence (*p* ≤ 0.0467) to support a detectable difference between YG1 and YG3 (37.62 ± 5.22%), as well as YG1 and YG5 (27.08 ± 5.41%). There was no detectable difference (*p* ≥ 0.9011) supporting an association for cattle with HS1 within YG3, YG4, or YG5. There was no evidence (*p* = 0.5543) to support a detectable difference in the proportion of cattle with HS2 between each YG category. One animal had a YG1, and this animal had an HS3. YG1 was not included in further comparisons between means due to underrepresentation. Cattle with HS3 comprised 35.97 ± 5.88% of the YG5 group, 29.87 ± 4.68% of YG3, 27.26 ± 4.48% of YG4, and 17.78 ± 5.74% of YG2. Although a heart score effect was identified (*p* = 0.0491), individual differences were not detected between yield grades after application of the post-hoc statistical adjustment. There was no detectable difference (*p* = 0.4973) between the proportion of cattle with HS4 between each USDA YG.

## 4. Discussion

Cattle with normal hooves represented the greatest proportion of hoof scores in this study (43%), but a majority of animals in this study had one or more hoof abnormalities. The most prevalent abnormality was an inward curve, followed by a wide toe and inward curve, followed by a corkscrew claw, a wide toe, a shovel, and an inward curve, then a shovel claw. Lee et al. (2018) [45] evaluated 65,600 finished cattle across six commercial slaughter facilities. Of those, 471 cattle had laminitis and 466 of the laminitic cattle had long toes (indicated in this study as shovel claw) [45]. Lee et al. (2018) [45] scored mobility on a four-point scale, where a score of 1 was normal mobility and scores 2–4 indicated mild mobility issues to severe mobility issues and/or non-ambulatory animals. Within the cattle that had laminitis, 423 animals had a mobility score of 2, 47 animals had a mobility score of 3, and 1 animal had a mobility score of 4. Most notably, none of the animals with laminitis had normal mobility scores. Terrell et al. (2017) [46] observed 6 large commercial feedlot operations in Kansas and Nebraska (N = 2432 animals). Of those, 99 animals had laminitis. The outcomes of those laminitis cases included 73 shipped to slaughter, 2 realized (sent for slaughter early), and 24 that died at the feedyard. In this group of natural-fed cattle, seven animals scored HOOF S, but an additional 17 animals had a shovel hoof present with other hoof abnormalities. Other hoof scores that were present in combinations included a shovel, wide toe, and inward curve; shovel and corkscrew; and corkscrew with a wide toe. In the study by Lee et al. (2018) [45], 130 animals had poor hoof conformation; 93% of those animals had a mobility score of 2, and 7% had a mobility score of 3. None of the animals with poor hoof conformation had a mobility score of 1 (normal mobility scores). A limitation to this study is that incidences of lameness were not evaluated. Based on previous research, however [45,46], there is a need to evaluate the relationship between hoof abnormalities and lameness in beef cattle.

Nine percent of cattle in this study had liver abscesses present. This natural program prohibited the use of fed ionophores, which is the leading mode of prevention for liver abscesses in the fed cattle industry [36,47,48]. The most recent national beef quality audit reported that 17.5% of fed steers and heifers had liver abscesses; this includes a variety of fed cattle in both conventional and natural programs in the United States [49]. Seven total animals in this study had a body wall adhesion, which can be a leading cause of reduced HCW in liver abscess cattle due to product loss with carcass trimming [50].

The proportion of cattle with normal hearts (HS1) in this study was 28.43%. This is less than what was observed in black angus steers in Wyoming (elevation: 2165 m) by Heffernan et al. (2020) [40], which had 37% of cattle with HS1, and Zuvich et al. (2025) [51], who observed 299 angus steers at slaughter originating from southeast Wyoming, of which 43% had HS1. Cattle with HS2 were less prevalent in this study than the 42% observed by Heffernan et al. (2020) [40] and the 39% observed by Zuvich et al. (2025) [51]. A larger proportion of cattle in this study had HS3 than Heffernan et al. (2020) [40] (16%) and Zuvich et al. (2025) [51] (15%). Two percent of cattle in this study had HS4, which was less than the 4.5% observed by Heffernan et al. (2020) [40] and the 3% observed by Zuvich et al. (2025) [51]. There were no cattle in this study earning HS5, but Zuvich et al. (2025) [51] did observe 3 animals with HS5. Overall, the natural cattle in this study had a greater prevalence of HS3 than other studies on similar populations of animals. The total days on feed was not available for the cattle in this study.

Of the cattle with multiple production diseases, the most common combination was cattle with hoof abnormalities and cardiac remodeling, representing 17.38% of animals. Both issues are conditions that compromise animal welfare in fed cattle [35]. There was no evidence to support a detectable association between hoof conformation and liver abscesses or heart score in this group of natural-fed cattle, which is similar to what was observed by Hamilton et al. (2026) [14], who recorded all three characteristics in conventional fed cattle (N = 398) at slaughter. Because the proportion of animals with each hoof score was not controlled in this study, some hoof types had a low number of evaluated animals. This limits some of the potential to compare relationships between scores. In this group, natural-fed cattle animals with no liver abscesses represented a greater proportion of those with no cardiac remodeling (HS1) than those with very mild cardiac remodeling (HS2). The total proportion of cattle with liver abscesses in this study was lower than the national average. It is possible that in cattle with a greater prevalence of liver abscesses, further relationships between production diseases could exist. Because of the close similarity between HS1 and HS2, observations on different populations of cattle are needed to understand a potential relationship. At this time, there are no additional studies that evaluate the incidence of liver abscesses relative to heart score.

Hot carcass weight for cattle with a shovel toe tended to be lower than for cattle with an inward curve or cattle with normal hooves. This contradicts what was reported by Hamilton et al. (2026) [14] for conventional cattle, in which HCW for cattle with a shovel toe was not different from cattle with an inward curve or normal hoof. Seixas et al. (2024) [52] evaluated 343 crossbred cattle from 103 different farms in Portugal and reported that HCW was not influenced by the presence of claw lesions in their group of cattle (asymmetrical claws, concave dorsal wall, corkscrew claw, dermatitis, double sole, heel horn erosion, fissures, interdigital hyperplasia, interdigital phlegmon, scissor claws, sole hemorrhage, swelling of coronet, ulcers, toe necrosis, thin sole, and white line abscess). Vollmar (2016) [53] surveyed feedlots in Colorado and Texas and determined that heavier cattle showed a significantly higher prevalence of front claw scissor-type abnormalities, representative of a corkscrew claw in this study. Cattle with larger HCW also had poorer mobility scores [53].

In this group of natural-fed cattle, there were no differences in average REA for each hoof score, which is consistent with the findings of Hamilton et al. (2026) [14] for conventional-fed cattle. This study observed no difference in average FT for each hoof score, consistent with the findings of Hamilton et al. (2026) [14] and Seixas et al. (2024) [52], who reported that the presence of claw lesions did not influence fat coverage in cattle.

Cattle with a corkscrew hoof represented more cattle in the Choice category than the Prime category. While not significant, this is the inverse of what was reported by Hamilton et al. (2026) [14] for conventional-fed cattle, where cattle with corkscrew hooves comprised 9.68% of those that are USDA Prime and 5.99% of those who were Choice. There was no further evidence to support a difference in the proportion of each of the remaining hoof scores within each USDA QG, which is consistent with the findings of Hamilton et al. (2026) [14].

Cattle with an inward curve comprised more of those with YG2 than YG3 or YG4 and tended to comprise more than those with YG5. Hamilton et al. (2026) [14] reported that HOOF I comprised the greatest prevalence of those with YG 5, followed by YG4, YG3, YG2, then YG1, although this difference was not significant. For the remaining hoof scores, there was no difference between USDA YG, which is consistent with Hamilton et al. (2026) [14].

For this group of natural-fed cattle, there was no difference in average HCW for each liver abscess score. These findings are corroborated by those of Hamilton et al. (2026) [14], as they reported no differences in average HCW for each liver abscess score for conventional-fed cattle. Grimes et al. (2024) [54] evaluated N = 1,537,532 carcasses from a beef database and reported that cattle with LIVER A+ and LIVER B had significantly lower HCW than cattle with LIVER A-. Herrick et al. (2023) [55] is in agreement with Grimes et al. (2024) [54] who reported that in fed Holsteins, carcasses with adhered and open liver abscesses had a lower HCW than carcasses without liver abscesses. Additionally, Torres et al. (2024) [56] performed a meta-analysis which concluded that animals with liver abscesses had a decrease in overall HCW. It is possible that these relationships did not exist in the population of natural-fed cattle in this study due to their low liver abscess prevalence. Herrick et al. (2023) [55] evaluated cattle with a prevalence of 44.38%, Grimes et al. (2024) [54] had a prevalence of 18.6–25.5%, and Torres et al. (2024) [56] evaluated previous publications focused on cattle with liver abscesses.

There was a detectable difference in the association between average REA and each liver abscess score for these natural-fed cattle, which is consistent with what was reported by Hamilton et al. (2026) [14] for conventionally fed cattle. The findings of Grimes et al. (2024) [54] contradict this, as they reported that cattle with LIVER A- had significantly greater loin muscle area (LMA) than cattle with LIVER A+, which in turn had greater LMA than LIVER B. Torres et al. (2024) [56] reported that animals with liver abscesses had a 1.21% reduction in average ribeye area relative to animals with no liver abscesses.

Grimes et al. (2024) [54] reported that cattle with LIVER B had significantly less FT than cattle with LIVER A- and LIVER A+, and cattle with CHF diagnosed from nutmeg liver had less FT than cattle with LIVER A- and LIVER A+. This difference was not observed in our study, however, nor was it observed in the study of conventional-fed cattle by Hamilton et al. (2026) [14] or in the meta-analysis by Torres et al. (2024) [56].

The previous literature is consistent with the findings in this study regarding the lack of evidence to support an association between liver abscesses and USDA QG and USDA YG. Grimes et al. (2024) [54] reported that marbling scores and USDA YG did not differ for liver scores A-, A+, and B in cattle diagnosed with CHF from nutmeg liver. Hamilton et al. (2026) [14] reported that there was no difference in the proportion of cattle with LIVER 0 in each USDA QG and USDA YG. Torres et al. (2024) [56] reported that the presence of liver abscesses had no effect on marbling score.

Cattle with HS3 had a greater HCW than cattle with HS1 or cattle with HS2, and their HCW did not differ from that of cattle with HS4. Hamilton et al. (2026) [14] scored hearts on a 3-point scale but reported similar results: cattle with mild cardiac remodeling had heavier carcasses than cattle with no remodeling and did not differ from those with severe remodeling. Torres-Quijada et al. (2024) [57] and Zuvich et al. (2025) [51] reported that HCW was 15 kg and 31.82 kg smaller, respectively, for HS4 than for HS1. These results indicate that heart scores may have differences in HCW. Grimes et al. (2024) [54] diagnosed cattle with CHF via the presence of nutmeg liver, and reported that those cattle with CHF had a significantly lower HCW than those with all other liver abscess scores. Heffernan et al. (2020) [40] contradict these results, as they found no difference in HCW between heart scores.

In this study, cattle with HS2 had a smaller observed average REA than cattle with HS1 or HS3, and were not different from cattle with HS4. Hamilton et al. (2026) [14] scored cardiac remodeling on a 3-point scale and reported that cattle with mild swelling tended to have the largest REA, followed by normal hearts, and lastly cattle with severe cardiac remodeling. Zuvich et al. (2025) [51] also described that there is a tendency for heart score to affect REA. Heffernan et al. (2020) [40] reported no differences in REA for each heart score in their study.

Cattle with HS4 tended to have less FT than HS2 and HS3, but were not different from HS1. Zuvich et al. (2025) [51] reported that there was a tendency for HS to affect back fat thickness. Three other studies [14,40,57] reported no differences in fat thickness for each heart score.

In this group of natural-fed cattle, there was no evidence to support a detectable difference in the proportion of cattle with each HS within each USDA QG. This is consistent with findings by Hamilton et al. (2026) [14], Heffernan et al. (2020) [40], and Zuvich et al. (2025) [51], who determined that there was no difference in their respective populations of cattle.

Cattle with normal hearts (HS1) comprised a greater proportion of YG2 than YG3 and YG5. Hamilton et al. (2026) [14] reported that cattle with normal hearts tended to comprise more of those in YG1 than YG3 or YG2. There was no difference in the proportion of HS4 for each USDA YG. This is consistent with Hamilton et al. (2026) [14] for severe cardiac remodeling, Heffernan et al. (2020) [40], who reported no difference in HS for each USDA YG, and Zuvich et al. (2025) [51], who reported that for all USDA YG, there was no difference in the proportion of each HS composing each category.

This study includes limitations related to the availability of backgrounding and genetic information from the source population of animals. It is not possible to understand causal relationships between production diseases in this cross-sectional observational study. Due to rapid line speed and study design, the front two hooves of each animal were evaluated. Future studies evaluating lameness and hind-limb conformation would be beneficial to understanding the impact of abnormal hoof conformation.

## 5. Conclusions

Fifty-seven percent of the *Bos taurus* natural-program fed cattle enrolled in this study (N = 1312) had one or more hoof abnormalities present at slaughter. Nine percent had liver abscesses, and 34% had heart scores of three or four on a five-point scale. Cattle with normal hearts had fewer liver abscesses than cattle with HS2 (mild changes). This study revealed no evidence to support an existing association between hoof score and liver score, or hoof score and heart score. Further relationships existed between specific hoof and heart scores and carcass characteristics. This study is limited by a lack of available backgrounding and genetic information but serves as an initial observation for potential relationships between heart score and liver score. Further research is warranted to evaluate genetic contributions to hoof abnormalities, liver abscesses, and cardiac remodeling, and the impact of backgrounding method on the development of production diseases.

## Figures and Tables

**Table 1:** Descriptive statistics for hot carcass weight (HCW), ribeye area (REA), and fat thickness (FT) for the sample population of natural-fed cattle (N = 1312).

	n	Mean	SD	Median	Minimum	Maximum
HCW (kg)	1312	464.85	53.44	460.03	319.82	654.59
REA (cm^2^)	1288	87.15	9.35	87.10	53.55	128.39
FT (cm)	946	3.93	0.54	3.9	2.3	5.8

**Table 2:** Proportion and 95% confidence intervals (CI) for each hoof score, liver score, and heart score for this sample of natural-fed cattle (N = 1312).

Attribute		% (Proportion)	95% CI
Hoof Score			
	Normal (HOOF N)	42.76 (561/1312)	40.06 to 45.49
	Corkscrew (HOOF C)	4.04 (53/1312)	3.04 to 5.25
	Inward Curve (HOOF I)	40.02 (525/1312)	37.35 to 42.72
	Shovel (HOOF S)	0.53 (7/1312)	0.21 to 1.10
	Shovel and Corkscrew (HOOF SC)	0.15 (2/1312)	0.02 to 0.55
	Shovel and Inward Curve (HOOF SI)	0.76 (10/1312)	0.004 to 1.40
	Shovel, Wide, and Inward Curve (HOOF SWI)	0.38 (5/1312)	0.12 to 0.89
	Wide and Corkscrew (HOOF WC)	0.08 (1/1312)	0.00 to 0.42
	Wide (HOOF W)	2.59 (34/1312)	1.80 to 3.60
	Wide and Inward Curve (HOOF WI)	8.69 (114/1312)	7.22 to 10.35
Liver Abscess Score			
	0 (Normal)	91.31 (1198/1312)	89.65 to 92.78
	A-	5.56 (73/1312)	4.39 to 6.95
	A	1.83 (24/1312)	1.16 to 2.71
	A+	0.76 (10/1312)	0.37 to 1.40
	B	0.53 (7/1312)	0.21 to 1.10
Heart Score			
	1	28.43 (373/1312)	26.00 to 30.96
	2	37.73 (495/1312)	35.10 to 40.41
	3	31.63 (415/1312)	29.12 to 34.22
	4	2.21 (29/1312)	1.49 to 3.16

**Table 3:** Proportion of cattle (N = 1312) with two or more production diseases present.

		% of Cattle in Subset (Proportion)
2 conditions present		22.41% (294/1312)
	Hoof Abnormality and Liver Abscess Present	3.43% (45/1312)
	Hoof Abnormality and Cardiac Remodeling Present	17.38% (228/1312)
	Liver Abscess and Cardiac Remodeling Present	1.60% (21/1312)
3 conditions present (Hoof Abnormality, Liver Abscess, and Cardiac Remodeling Present)		1.22% (16/1312)

**Table 4:** Mean and SE for the proportion of each hoof score present within each liver score for this sample of natural-fed cattle (N = 1312).

	Liver Score ^2^												
	0 (No Abscess) ^1^			A− ^2^			A ^2^			A+/B ^2^			
Hoof Score ^1^	EM Means (%)	Observed Proportion	SEM	EM Means (%)	Observed Proportion	SEM	EM Means (%)	Observed Proportion	SEM	EM Means (%)	Observed Proportion	SEM	Overall *p*-Value
Normal (N)	35.32	508/1198	4.78	54.45	30/73	11.65	39.43	15/24	12.67	34.68	8/17	7.14	0.3204
Corkscrew (C)	3.84	46/1198	0.56	4.17	6/73	4.08	5.88	1/24	5.71	8.22	1/17	3.22	0.3462
Inward Curve (I)	40.48	485/1198	1.42	16.67	30/73	7.61	35.29	4/24	11.59	41.10	6/17	5.76	0.1630
Shovel (S) ^3^	0.26	22/1198	54.89	0.00	2/73	0.00	0.00	0/24	0.00	0.35	0/17	74.85	-
Wide (W)	2.67	32/1198	0.47	4.17	1/73	4.08	0.00	1/24	0.00	1.37	0/17	1.36	-
Wide and Inward Curve (WI)	8.77	105/1198	0.82	12.50	4/73	6.75	11.76	3/24	7.81	5.48	2/17	2.66	-

^1^ Hoof scores are combined into the following categories: Normal (HOOF N), Corkscrew (HOOF C, HOOF CW), Inward Curve (HOOF I), Shovel (HOOF S, HOOF SC, HOOF SI, HOOF SWI), Wide (HOOF W), Wide and Inward Curve (HOOF WI). The following categories did not meet the minimum requirement of >5 per group for comparisons between means: HOOFC and LIVER A, LIVER A+, HOOF I and LIVER A, HOOF S and LIVER A-, LIVER A, LIVER A+, HOOF W and LIVER A-, LIVER A, LIVER A+, HOOF WI for LIVER A-, LIVER A, LIVER A+. ^2^ On cases where there is an interaction effect, the rounding for adjusted means results in a deviation from a total of 100% within a column. ^3^ SEM Values for HOOF S are reflective of a low representation of cattle with HOOF S relative to the study population. These values are descriptive only, and EM Means from those hoof scores are not included in the analysis.

**Table 5:** Mean and SE for the proportion of each hoof score present within each heart score in one group of natural-fed cattle (N = 1312).

	Heart Score ^2^												
	1			2			3			4			
Hoof Score ^1^	EM Means (%)	Observed Proportion	SEM	EM Means (%)	Observed Proportion	SEM	EM Means (%)	Observed Proportion	SEM	EM Means (%)	Observed Proportion	SEM	Overall *p*-Value
Normal (N)	35.06	153/373	5.07	34.44	208/495	5.08	36.51	185/415	5.23	44.22	15/29	10.44	0.6982
Corkscrew (C)	4.56	17/373	1.08	3.03	15/495	0.77	5.30	22/415	1.10	0.00	0/29	0.00	0.3944
Inward Curve (I)	39.68	148/373	2.53	42.83	212/495	2.22	37.11	154/415	2.37	37.93	11/29	9.01	0.3682
Shovel (S)	1.07	4/373	0.53	1.41	7/495	0.53	2.89	12/415	0.82	3.45	1/29	3.39	0.2212
Wide (W)	3.49	13/373	0.95	2.22	11/495	0.66	2.41	10/415	0.75	0.00	0/29	0.00	0.6979
Wide and Inward Curve (WI)	10.19	38/373	1.57	8.49	42/495	1.25	7.71	32/415	1.31	6.90	2/29	4.71	0.6390

^1^ Hoof scores are combined into the following categories: Normal (HOOF N), Corkscrew (HOOF C, HOOF CW), Inward Curve (HOOF I), Shovel (HOOF S, HOOF SC, HOOF SI, HOOF SWI), Wide (HOOF W), Wide and Inward Curve (HOOF WI). The following categories did not meet the minimum requirement of >5 per group for comparisons between means: HS1 for HOOFS, HS4 for HOOF C, HOOF W, and HOOF WI. ^2^ In cases where there is an interaction effect, the rounding for adjusted means results in a deviation from a total of 100% within a column.

**Table 6:** Mean and SE for the proportion of each liver score present within each heart score for one group of natural-fed cattle (N = 1312).

	Heart Score ^3^												
	1			2			3			4			
Liver Score ^2^	EM Means (%)	Observed Proportion	SEM	EM Means (%)	Observed Proportion	SEM	EM Means (%)	Observed Proportion	SEM	EM Means (%)	Observed Proportion	SEM	Overall *p*-Value
0 (No Abscess)	94.37 ^a^	352/373	1.19	88.69 ^bc^	439/495	1.42	91.57 ^ac^	380/415	1.36	93.10 ^ab^	27/29	4.71	0.0355
A-	4.02	15/373	1.02	7.68	38/495	1.20	4.82	20/415	1.05	0.00	0/29	0.00	0.1119
A	0.80	3/373	0.46	2.02	10/495	0.63	2.17	9/415	7.15	6.90	2/29	4.71	0.1287
A+/B ^1^	0.80	3/373	0.46	1.62	8/495	0.57	1.45	6/415	0.59	0.00	0/29	0.00	0.7766

^1^ Liver scores A+ and B were combined for these analyses. ^2^ The following categories did not meet the minimum requirement of >5 per group for comparisons between means. Therefore, these results are descriptive only in nature: LIVER A- and HS4, LIVER A and HS1, HS4, and LIVER A+ and HS1, HS4. ^a,b,c^ Superscripts that differ within a row identify significant differences between heart scores across each liver abscess score (*p* ≤ 0.05). ^3^ In cases where there is an interaction effect, the rounding for adjusted means results in a deviation from a total of 100% within a column.

**Table 7:** Mean and SE for each hot carcass weight (HCW) ribeye area (REA) and fat thickness (FT) for each hoof score for one group of natural-fed cattle (N = 1312).

	Independent Variable								
	HCW (kg)			REA (cm^2^)			FT (cm)		
Hoof Score ^1^	n	LS Means	SE	n	LS Means	SE	n	LS Means	SE
Normal (N)	561	467.04 ^x^	2.25	555	86.89	0.40	401	3.95	0.03
Corkscrew (C)	54	456.92 ^xy^	7.25	53	86.03	1.28	36	3.87	0.09
Inward Curve (I)	525	466.79 ^x^	2.33	511	87.69	0.41	387	3.93	0.03
Shovel (S) ^2^	24	435.99 ^y^	10.87	23	87.10	1.95	16	3.94	0.13
Wide (W)	34	452.61 ^xy^	9.14	33	85.47	1.63	25	3.74	0.11
Wide and Inward Curve (WI)	114	458.66 ^xy^	5.00	113	86.99	0.88	81	3.88	0.06
Overall *p*-Value	0.0219			0.5476			0.4480		

^1^ Hoof scores are combined into the following categories: Normal (HOOF N), Corkscrew (HOOF C, HOOF CW), Inward Curve (HOOF I), Shovel (HOOF S, HOOF SC, HOOF SI, HOOF SWI), Wide (HOOF W), Wide and Inward Curve (HOOF WI). ^2^ HOOF S was not included in comparisons between means for REA and FT as it did not meet distribution requirements for analysis. ^x,y^ Superscripts that differ within a column identify tendencies between carcass characteristics across hoof scores (0.05 ≤ *p* < 0.10).

**Table 8:** Mean and SE for the proportion of each hoof, liver, and heart score present within each reported quality grade (QG) for one group of natural fed cattle (N = 1312).

	Quality Grade ^3^									Overall *p*-Value
	Prime			Choice			Select ^4^			
Hoof Score ^1,4^	EM Means (%)	Observed Proportion	SEM	EM Means (%)	Observed Proportion	SEM	EM Means (%)	Observed Proportion	SEM	
Normal (N)	36.45	161/407	4.92	32.93	389/886	4.98	52.84	11/19	12.81	0.1464
Corkscrew (C)	2.94 ^b^	28/407	0.57	6.88 ^a^	26/886	1.26	0.00	0/19	0.00	0.0061
Inward Curve (I)	40.63	160/407	1.65	39.31	360/886	2.42	26.32	5/19	10.10	0.4358
Shovel (S)	0.44	5/407	34.47	0.23	18/886	17.85	1.10	1/19	85.20	0.2670
Wide (W)	2.48	12/407	0.52	2.95	22/886	0.84	0.00	0/19	0.00	0.8885
Wide and Inward Curve (WI)	8.01	41/407	0.91	10.07	71/886	1.49	10.53	2/19	7.04	0.4569
Liver Score ^2,4^										
0 (No Abscess)	91.40	372/407	1.39	91.42	810/886	0.94	84.21	16/19	8.37	0.5548
A-	6.14	25/407	1.19	5.31	47/886	0.75	5.26	1/19	5.12	-
A	1.23	5/407	0.55	1.92	17/886	0.46	10.53	2/19	7.04	-
A+/B	1.35	5/407	0.39	1.23	12/886	0.55	0.00	0/19	0.00	-
Heart Score ^4^										
1	36.75	114/407	5.34	38.31	254/886	5.06	31.23	5/19	12.41	0.7701
2	37.84	154/407	2.40	37.81	335/886	1.63	31.58	6/19	10.66	0.8571
3	28.00	132/407	4.38	30.33	275/886	4.87	41.40	8/19	12.49	0.3453
4	1.72	7/407	0.64	2.48	22/886	0.52	0.00	0/19	0.02	0.6930

^1^ Hoof scores are combined into the following categories: Normal (HOOF N), Corkscrew (HOOF C, HOOF CW), Inward Curve (HOOF I), Shovel (HOOF S, HOOF SC, HOOF SI, HOOF SWI), Wide (HOOF W), Wide and Inward Curve (HOOF WI). ^2^ Liver scores A+ and B were combined for these analyses. ^3^ In cases where there is an interaction effect, the rounding for adjusted means results in a deviation from a total of 100% within a column. ^4^ The following categories did not meet the minimum requirement of >5 per group for comparisons between means. Therefore, these results are descriptive only in nature: USDA QG Select and HOOF C, HOOF S, HOOF W, HOOF WI, LIVER A-, LIVER A, LIVER A+, and HS4. ^a,b^ Superscripts that differ within a row identify significant differences between heart scores across each liver abscess score (*p* ≤ 0.05).

**Table 9:** Mean and SE for the proportion of each hoof, liver, and heart score present within each USDA yield grade (YG) for one group of natural fed cattle (N = 1312).

	Yield Grade ^3,4^															Overall *p*-Value
	1			2			3			4			5			
Hoof Score ^1^	EM Means (%)	Observed Proportion	SEM	EM Means (%)	Observed Proportion	SEM	EM Means (%)	Observed Proportion	SEM	EM Means (%)	Observed Proportion	SEM	EM Means (%)	Observed Proportion	SEM	
Normal (N)	100.00	1/1	0.45	30.31	21/58	7.18	34.80	195/474	4.97	37.60	255/566	5.15	33.42	89/213	5.58	0.6610
Corkscrew (C)	100.00	0/1	0.00	0.00	0/58	0.00	4.01	19/474	0.98	5.12	29/566	0.85	2.82	6/213	1.11	0.7131
Inward Curve (I)	0.00	0/1	0.31	55.17 ^a^	32/58	6.54	40.72 ^b^	193/474	2.25	36.93 ^b^	209/566	2.03	42.72 ^ab^	91/213	33.92	0.0794
Shovel (S)	0.00	0/1	0.05	0.28	1/58	22.04	0.43	11/474	33.91	0.38	10/566	29.57	0.23	2/213	18.48	0.9508
Wide (W)	0.00	0/1	0.31	1.72	1/58	1.71	2.74	13/474	0.75	2.47	14/566	0.65	2.82	6/213	1.11	0.9906
Wide and Inward Curve (WI)	0.00	0/1	0.00	5.17	3/58	2.91	9.07	43/474	1.32	8.66	49/566	1.18	8.92	19/213	1.95	0.9126
Liver Score ^2^																
0 (No Abscess)	0.00	0/1	0.00	94.83	55/58	2.91	90.30	428/474	1.36	91.87	520/566	1.15	91.55	195/213	1.91	0.7837
A-	100.00	1/1	0.31	1.72	1/58	1.71	6.12	29/474	1.11	5.48	31/566	0.96	5.16	11/213	1.52	0.7737
A	0.00	0/1	0.31	1.72	1/58	1.71	2.11	10/474	0.66	1.41	8/566	0.50	2.35	5/213	1.04	0.9009
A+/B	0.00	0/1	0.31	1.72	1/58	1.71	1.48	7/474	0.55	1.24	7/566	0.46	0.94	2/213	0.66	0.9802
Heart Score																
1	0.00	0/1	0.49	57.08 ^a^	27/58	8.17	37.62 ^b^	141/474	5.22	38.03 ^ab^	165/566	5.27	27.08 ^b^	40/213	5.41	0.0032
2	0.00	0/1	0.31	29.31	17/58	5.98	36.29	172/474	2.21	39.58	224/566	2.06	38.50	82/213	3.33	0.5543
3 ^5^	100.00	1/1	0.43	17.78	11/58	5.74	29.87	149/474	4.68	27.26	168/566	4.48	35.97	86/213	5.88	0.0491
4	0.00	0/1	0.31	5.17	3/58	2.91	2.53	12/474	0.72	1.59	9/566	0.53	2.35	5/213	1.04	0.4973

^1^ Hoof scores are combined into the following categories: Normal (HOOF N), Corkscrew (HOOF C, HOOF CW), Inward Curve (HOOF I), Shovel (HOOF S, HOOF SC, HOOF SI, HOOF SWI), Wide (HOOF W), Wide and Inward Curve (HOOF WI). ^2^ Liver scores A+ and B were combined for these analyses. ^a,b^ Superscripts that differ within a row identify significant differences between Yield Grades across each heart score (*p* ≤ 0.05). ^3^ In cases where there is an interaction effect, the rounding for adjusted means results in a deviation from a total of 100% within a column. ^4^ The following categories did not meet the minimum requirement of >5 per group for comparisons between means. Therefore, these results are descriptive only in nature: USDA YG1 for all production disease scores, USDA YG 2 for HOOF C, HOOF S, HOOF W, HOOF WI, LIVER A-, LIVER A, LIVER A+, HS 4, USDA YG 5 for HOOF S, LIVER A+. ^5^ Differences between EM Means for heart score 3 were not detected (*p* > 0.05) between yield grades after application of the post-hoc statistical adjustment although a main effect was identified (*p* = 0.0491).

**Table 10:** Mean and SE for hot carcass weight (HCW), ribeye area (REA), and fat thickness (FT) for each liver score for one group of natural-fed cattle (N = 1312).

	Independent Variable								
	HCW (kg) ^2^			REA (cm^2^)			FT (cm)		
Liver Score	n	LS Means	SE	n	LS Means	SE	n	LS Means	SE
0 (No Abscess)	1198	448.24	4.44	1176	87.21	0.27	868	3.93	0.02
A-	73	-	-	71	87.54	1.11	51	3.93	0.08
A	24	-	-	24	83.33	1.91	16	3.92	0.13
A+/B ^1^	17	-	-	17	86.72	2.27	11	3.95	0.16
Overall *p*-Value	0.4764			0.2404			0.9994		

^1^ Liver scores A+ and B were combined for these analyses. ^2^ For HCW, means are not reported for LIVER A-, LIVER A, and LIVER A+/B due to underrepresentation between producers.

**Table 11:** Mean and SE for hot carcass weight (HCW), ribeye area (REA), and fat thickness (FT) for each heart score for one group of natural-fed cattle (N = 1312).

	Independent Variable								
	HCW (kg)			REA (cm^2^)			FT (cm)		
Heart Score	n	LS Means	SE	n	LS Means	SE	n	LS Means	SE
1	373	457.34 ^b^	2.74	369	87.81 ^a^	0.49	269	3.87 ^xz^	0.03
2	495	460.76 ^b^	2.38	481	86.06 ^b^	0.43	348	3.96 ^x^	0.03
3	415	476.16 ^a^	2.60	409	87.71 ^a^	0.46	306	3.96 ^x^	0.03
4	29	469.54 ^ab^	9.82	29	88.85 ^ab^	1.73	23	3.69 ^yz^	0.11
Overall *p*-Value	<0.0001			0.0125			0.0161		

^a,b^ Superscripts that differ within a column identify significant differences between carcass characteristics within heart scores across placements (*p* ≤ 0.05). ^x,y,z^ Superscripts that differ within a column identify tendencies between carcass characteristics across hoof scores (0.05 ≤ *p* < 0.10).

## Data Availability

The raw data supporting the conclusions of this article will be made available by the authors on request.

## Abbreviations

The following abbreviations are used in this manuscript:

CHF	Congestive Heart Failure
CFR	Code of Federal Regulations
HOOF C	Corkscrew
CI	Confidence Interval
FSIS	Food Safety Inspection Service
FT	Fat Thickness
HCW	Hot Carcass Weight
HOOF I	Inward Curve
IACUC	Institutional Animal Care and Use Committee
HOOF N	Normal
NASS	National Agricultural Statistics Service
BQA	Beef Quality Audit
PAP	Pulmonary Arterial Pressure
QG	Quality Grade
REA	Ribeye Area
HOOF S	Shovel
HOOF SC	Shovel and Corkscrew
HOOF SI	Shovel and Inward Curve
HOOF SWI	Shovel, Wide Toe, and Inward Curve
USDA	United States Department of Agriculture
HOOF CW	Corkscrew and Wide Toe
HOOF W	Wide Toe
HOOF WI	Wide Toe and Inward Curve
YG	Yield Grade
